# Chemical Characterization and In Vitro Biological Activity of Edible Desert Truffle *Tirmania pinoyi* CCASU-TP-03

**DOI:** 10.3390/ijms27219925

**Published:** 2026-11-05

**Authors:** Shaza M. Elhusseiny, Salwa Abougalambou, Ahmed M. El-Dessouki, Khaled M. Aboshanab, Hesham Ali El Enshasy, Mohamed H. Al-Agamy, Khalid Alyahya, Samar Ibrahim

**Affiliations:** 1 Department of Microbiology and Immunology, Faculty of Pharmacy, Ahram Canadian University (ACU), Giza 12566, Egypt; shaza.ahmed@acu.edu.eg; 2 Department of Pharmacy Practice, Pharmacy College, Ahram Canadian University (ACU), Giza 12451, Egypt; salwa.selim@acu.edu.eg; 3 Department of Pharmacology and Toxicology, Faculty of Pharmacy, Ahram Canadian University (ACU), Giza 12566, Egypt; ahmed.desoky@acu.edu.eg; 4 Department of Microbiology and Immunology, Faculty of Pharmacy, Ain Shams University, Cairo 11566, Egypt; 5 Innovation Centre in Agritechnology for Advanced Bioprocessing (ICA), Universiti Teknologi Malaysia (UTM), Pagoh Campus, Pagoh 84600, Malaysia; hesham@utm.my; 6 Department of Pharmaceutics, College of Pharmacy, King Saud University, Riyadh 11451, Saudi Arabia; 7 Department of Pharmacy Practice and Clinical Pharmacy, Faculty of Pharmacy, Galala University, Galala 43511, Egypt

**Keywords:** *Tirmania pinoyi*, desert truffle, antioxidant activity, antimicrobial activity, anticancer activity, anti-inflammatory activity, iNOS

## Abstract

*Tirmania pinoyi* is an edible desert truffle valued for its nutritional properties; however, information regarding its biological activity and chemical composition is still limited. The current investigation sought to describe the chemical makeup of *T. pinoyi* CCASU-TP-03 extract and assess its biological properties. Molecular identification based on 25S rDNA and ITS1–5.8S–ITS2 sequence analysis placed CCASU-TP-03 within the *T. pinoyi* lineage, supporting the morphological identification. The extract was characterized by its chemical composition, while its antioxidant, antimicrobial, cytotoxic, and anti-inflammatory activities were evaluated using DPPH/ORAC, broth microdilution, SRB, and Griess assays, respectively. Western blotting was used to assess iNOS protein expression. The methanolic extract contained total carbohydrate content expressed as glucose equivalents (354.25 ± 7.71 mg glucose equivalents/g extract), total phenolics (72.57 ± 0.12 mg GAE/g extract), total flavonoids (9.83 ± 0.02 mg RE/g extract), and protein (11.12 ± 0.57 mg/g extract). Targeted qualitative HPLC-PDA profiling detected selected phenolic and flavonoid constituents, including gallic acid, isoferulic acid, ellagic acid, quercetin, and rutin. The *T. pinoyi* CCASU-TP-03 extract exhibited measurable antioxidant capacity in both DPPH and ORAC assays. Antimicrobial evaluation demonstrated inhibitory activity against several of the tested bacterial and yeast strains, whereas no activity was detected against *Aspergillus niger* under the tested conditions. Cytotoxicity screening showed the greatest reduction in viability in CaCo-2 cells, whereas PANC-1, HepG2, and HCT-116 cells maintained comparatively high viability over the investigated concentration range. The extract showed measurable inhibition of LPS-induced NO production accompanied by reduced iNOS protein expression. In conclusion, the findings demonstrate measurable antioxidant capacity, inhibitory activity against several tested microorganisms, differential cytotoxic effects among the tested cancer cell lines, and inhibition of NO production accompanied by reduced iNOS protein expression. Further studies are required to isolate the respective pure metabolites followed by preclinical evaluation for potential use in humans.

## 1. Introduction

Edible mushrooms are widely recognized as valuable food sources in many cultures owing to their distinctive flavor and nutritional value [1]. Desert truffles have recently attracted considerable scientific interest owing to their health benefits and their richness in diverse bioactive constituents [2,3]. Investigation of the potential use of desert truffles and their prospective uses in the culinary, pharmaceutical, and nutraceutical industries has increased due to their naturally occurring compounds [4]. Previous investigations of *T. pinoyi* have described its nutritional composition, antimicrobial properties, metabolite profiles, and selected biological activities; therefore, the novelty of the present study lies not in the first evaluation of the species but in the integrated characterization of the molecularly authenticated CCASU-TP-03 isolate using complementary chemical and in vitro assays.

Nevertheless, the biological properties of many edible desert truffle species remain insufficiently explored, particularly those growing under different environmental conditions that may influence their chemical composition and bioactivity [4].

Truffles are subterranean fungi belonging to the phylum *Ascomycota* and the order *Pezizales*. They comprise several genera, including *Tuber*, *Terfezia*, and *Tirmania*, with *Tuber* species being the most commercially valuable, whereas *Tirmania* and *Terfezia* are the predominant edible desert truffles distributed throughout North Africa, the Middle East, and other dry regions of the world [5]. The diverse bioactive constituents identified in truffles have stimulated growing interest in their potential biological activities. While several studies have investigated health-related properties in different truffle species, the biological potential of many edible desert truffles, including *T. pinoyi*, remains insufficiently characterized.

In addition to their nutritional benefits, mushrooms are known to be abundant in phenolic compounds. These bioactive constituents may be associated with the benefits of mushrooms for health and have therefore been widely investigated. Phenolic compounds can scavenge free radicals by transferring electrons or donating hydrogen atoms and may also reduce oxidative stress by chelating transition metals and modulating oxidative enzymes [6]. Consequently, they are considered important contributors to the mushrooms’ antioxidant capacity, with several studies reporting positive correlations between total phenolic content and antioxidant activity [7].

Besides phenolic compounds, mushrooms are a valuable source of flavonoids, proteins, carbohydrates, polysaccharides, and additional bioactive metabolites that may contribute to their biological properties [8,9]. Through various processes, these naturally occurring chemicals have been investigated for potential anticancer, anti-inflammatory, and antibacterial qualities, including the inhibition of microbial growth, modulation of inflammatory mediators, and suppression of cancer cell proliferation [10,11,12].

Previous studies on desert truffles have reported diverse nutritional and bioactive constituents, including phenolic compounds, flavonoids, polysaccharides, proteins, and other metabolites, which may contribute to their biological properties. Related species of *Tirmania* and other desert truffles, such as *Tirmania nivea* and *Terfezia boudieri*, have been investigated for antioxidant, antimicrobial, anti-inflammatory, and other biological activities [13]. However, the available information is considerably more limited for *T. pinoyi*, particularly regarding the relationship between its biochemical composition and its biological activities [5]. Moreover, comprehensive evaluations combining chemical characterization with antimicrobial, antioxidant, cytotoxic, and anti-inflammatory assays, along with investigations of iNOS expression, remain limited. These gaps provided the rationale for the present study.

Despite the growing interest in the bioactive constituents of edible mushrooms, previous studies have reported nutritional characteristics and biological activities of *T. pinoyi* and related desert truffles. However, integrated characterization of a molecularly authenticated *T. pinoyi* isolate combining chemical quantification, targeted phenolic/flavonoid profiling, antioxidant and antimicrobial evaluation, cytotoxicity screening, and assessment of the NO/iNOS response remains limited. Accordingly, this study aimed to characterize the methanolic extract of *T. pinoyi* CCASU-TP-03 by measuring total carbohydrates, protein, phenolics, and flavonoids and by targeted HPLC-PDA analysis, and to assess its antioxidant, antimicrobial, cytotoxic, and LPS-induced NO/iNOS-related effects in vitro.

## 2. Results

### 2.1. Collection and Identification of Truffle Isolate

**Figure 1:**
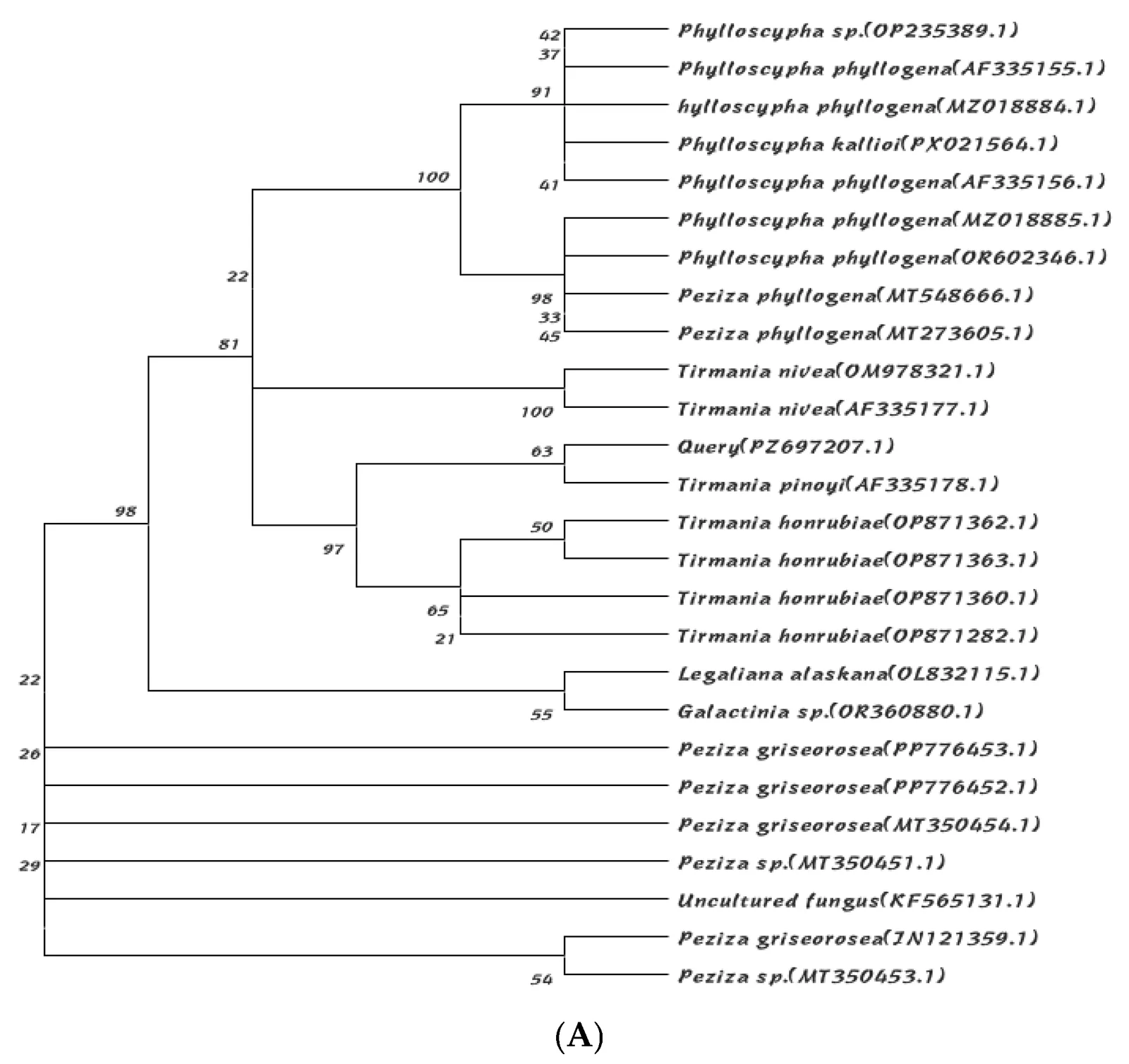

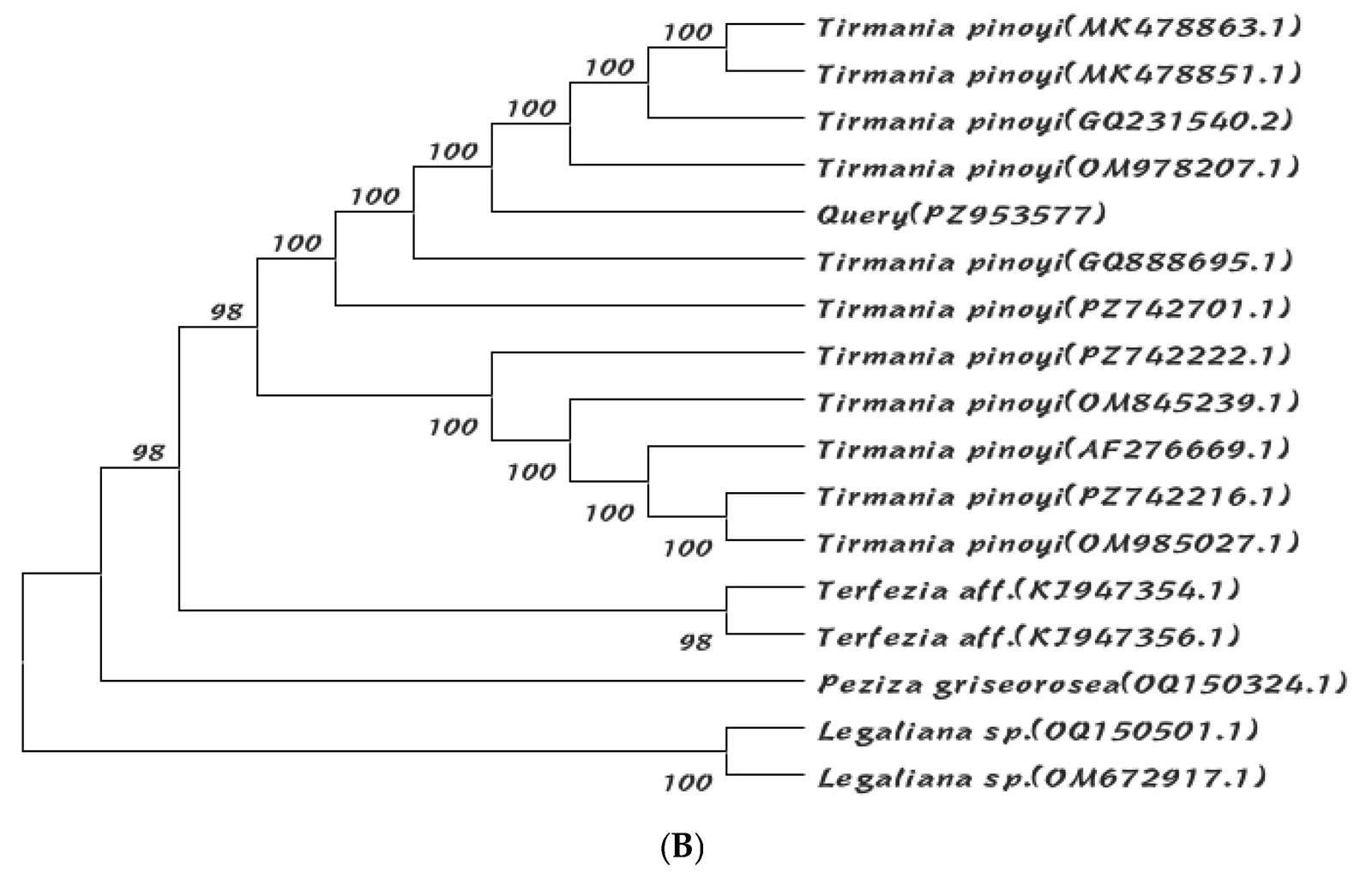
Phylogenetic tree of edible tested mushroom CCASU-TP-03 (Query); (**A**): large 25S ribosomal RNA subunit (NCBI accession: PZ697207.1); (**B**): ITS1–5.8S rRNA–ITS2 subunit (internal transcribed spacer 1 and 5.8S ribosomal RNA gene, and internal transcribed spacer 2; NCBI accession: PZ953577). Branch support values represent bootstrap percentages based on 1000 replicates.

Truffle samples were collected from the deserts of Jizan City, Saudi Arabia, morphologically characterized, and deposited at the Ain Shams University Culture Collection under voucher Nr. CCASU-TP-03. The large 25S ribosomal RNA gene sequence analysis and ITS1–5.8S rRNA–ITS2 region analysis revealed the identity of the samples as *T. pinoyi* as depicted in Figure 1A,B. However, the identification relies mainly on the ITS tree, as depicted in Figure 1B, since in tree A, the query groups with *T. pinoyi* with a bootstrap of only 63. The 25S rDNA sequence and ITS1–5.8S–ITS2 sequence were deposited in the NCBI GenBank database under accession numbers PZ697207 and PZ953577, respectively. The respective phylogenetic trees, as displayed in Figure 1A,B, were constructed using the maximum likelihood method with standard bootstrap in MEGA12 to demonstrate how closely the acquired sequence resembles the reference sequences in the genome database.

### 2.2. Chemical Analysis of T. pinoyi CCASU-TP-03 Extract

The chemical characterization of *T. pinoyi* CCASU-TP-03 extract revealed total carbohydrate content expressed as glucose equivalents at 354.25 ± 7.71 mg glucose equivalents/g extract, total phenolic content of 72.57 ± 0.12 mg GAE/g extract, total flavonoid content of 9.83 ± 0.02 mg RE/g extract, and total protein content of 11.12 ± 0.57 mg/g extract (Table 1). These measurements represent independently determined chemical parameters and are therefore reported using their respective analytical units and equivalents rather than as percentages of the total extract.

### 2.3. Targeted HPLC-PDA Analysis of Selected Phenolic and Flavonoid Compounds

Targeted HPLC-PDA analysis was performed to characterize selected phenolic and flavonoid compounds in the *T. pinoyi* CCASU-TP-03 extract. Eleven prespecified phenolic/flavonoid standards were screened by targeted HPLC-PDA, of which five were detected under the applied analytical conditions (Table 2). This targeted analysis does not represent comprehensive metabolomic characterization of the crude extract. Among the compounds included in the targeted analysis, gallic acid was detected at the highest concentration, followed by ellagic acid and isoferulic acid, whereas quercetin and rutin were also detected. Catechin, chlorogenic acid, caffeic acid, hesperidin, kaempferol, and apigenin were not detected under the applied analytical conditions. These findings provide a targeted profile of the selected compounds investigated in the present study and do not represent an exhaustive characterization of the extract metabolome. Targeted HPLC-PDA analysis was performed to screen selected phenolic and flavonoid compounds in the *T. pinoyi* CCASU-TP-03 extract. Compound assignment was based on comparison of chromatographic retention times and PDA spectral characteristics with those of the corresponding reference standards. Figure S1 displays the targeted HPLC-PDA analysis of selected phenolic and flavonoid compounds for the standard, and *T. pinoyi* CCASU-TP-03 extract.

### 2.4. Antimicrobial Activity

The antimicrobial activity of *T. pinoyi* extract varied among the tested microorganisms in Table 3*. B. subtilis* showed the highest susceptibility, with an MIC of 3.125 mg/mL, followed by *C. albicans* at 6.25 mg/mL. MIC values of 12.5 mg/mL were observed against *S. aureus* and *P. vulgaris*, while *E. coli* exhibited lower susceptibility with an MIC of 25 mg/mL. No inhibitory activity was detected against *A. niger* at the tested concentrations. Overall, the extract exhibited relatively weak antimicrobial activity under the experimental conditions.

### 2.5. Antioxidant Activity

**Figure 2:**
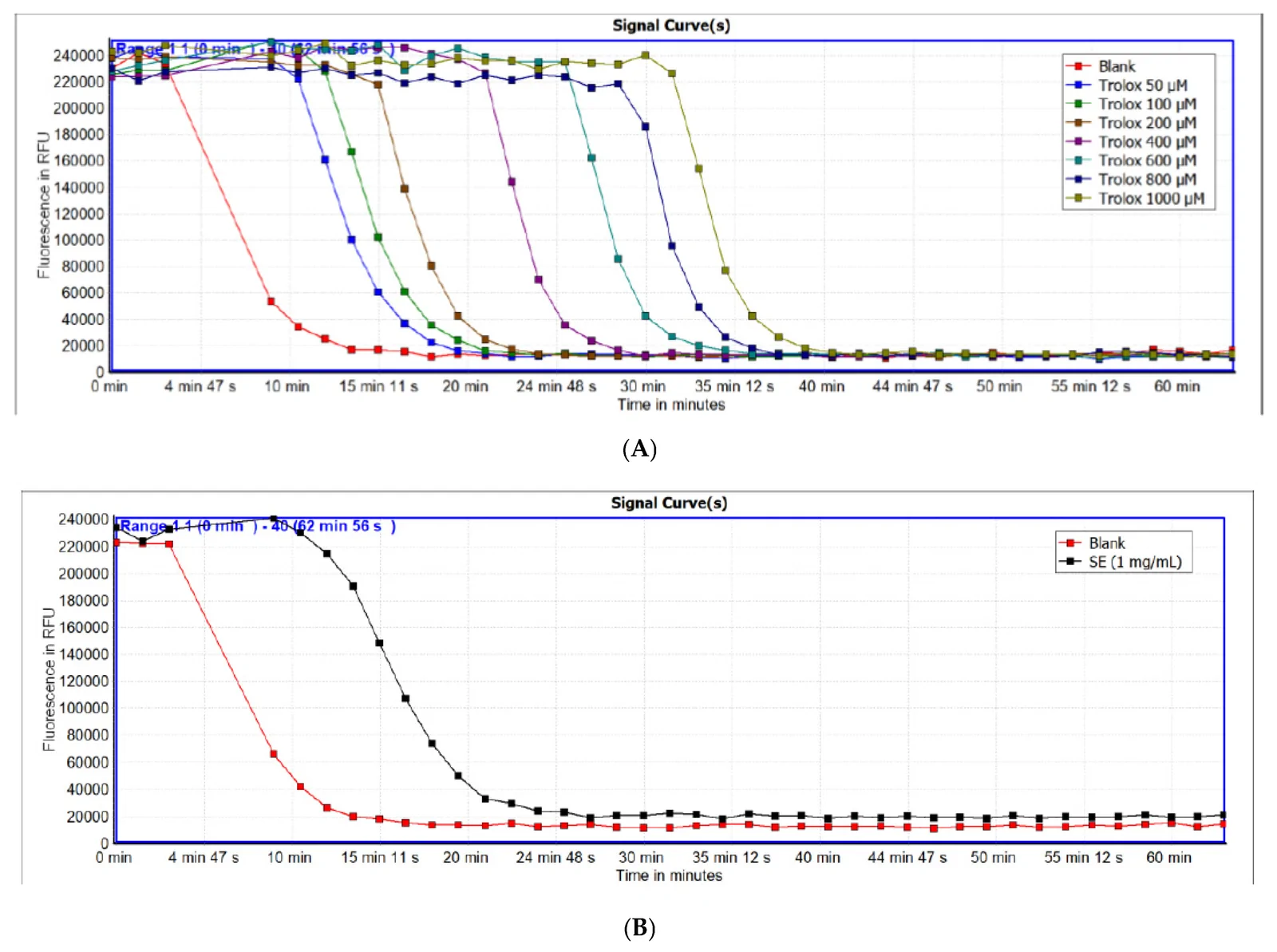
ORAC assay (**A**) The signal curves representing various concentrations of Trolox and a blank sample illustrate the decay of fluorescein at differing Trolox concentrations. (**B**) Signal curve of *T. pinoyi* CCASU-TP-03 extract and blank indicating the decay of fluorescein upon applying the samples.

The antioxidant potential of *T. pinoyi* CCASU-TP-03 extract was measured using both DPPH and ORAC assays in Table 4. In the DPPH assay, the extract demonstrated a concentration-dependent ability to scavenge free radicals with an IC_50_ value of 1070 ± 1.03 μg/mL. Furthermore, the ORAC assay demonstrated an antioxidant capacity of 231.12 ± 26.86 μmol TE/mg extract, indicating the ability of *T. pinoyi* CCASU-TP-03 extract to scavenge peroxyl radicals. As illustrated in Figure 2B, antioxidant capacity was calculated from the integrated area under the fluorescence-decay curve relative to the Trolox calibration curve. The 1,332,695 AUC values obtained for the extract were converted to Trolox equivalents using the calibration equation (y = 3526.176x + 527,521.245) (R^2^ = 0.997). Following normalization to the amount of extract analyzed, the ORAC value was 231.12 ± 26.86 μM Trolox equivalents (TE)/mg extract. Collectively, these findings confirm that *T. pinoyi* CCASU-TP-03 possesses measurable antioxidant activity, which may be attributed, at least in part, to its phenolic constituents identified by HPLC analysis. However, the relatively high DPPH IC_50_ value indicates limited radical-scavenging potency under the tested conditions.

### 2.6. Anti-Inflammatory Activity

The anti-inflammatory properties of *T. pinoyi* CCASU-TP-03 extract were evaluated through the Griess assay. As shown in Table 5, the extract inhibited LPS-induced nitric oxide (NO) production, particularly at higher concentrations. NO inhibition remained minimal and statistically indistinguishable at 31.25 and 62.5 μg/mL, followed by greater inhibition at higher concentrations, with the most pronounced effect observed at 500 μg/mL (40.23 ± 1.82%). To exclude the possibility that the observed reduction in NO production was attributable to reduced cell viability, the viability of RAW264.7 cells was assessed using the SRB assay at the corresponding extract concentrations. Cell viability was 101.65 ± 1.69%, 100.45 ± 0.88%, 100.01 ± 2.09%, 99.94 ± 1.28%, and 97.50 ± 1.72% at 31.25, 62.5, 125, 250, and 500 μg/mL, respectively. Notably, cell viability remained 97.50 ± 1.72% at 500 μg/mL, whereas NO production was inhibited by 40.23 ± 1.82%, supporting the conclusion that the observed reduction in NO was not attributable to an appreciable loss of RAW264.7 cell viability.

### 2.7. Effect of T. pinoyi CCASU-TP-03 Extract on iNOS Protein Expression

**Figure 3:**
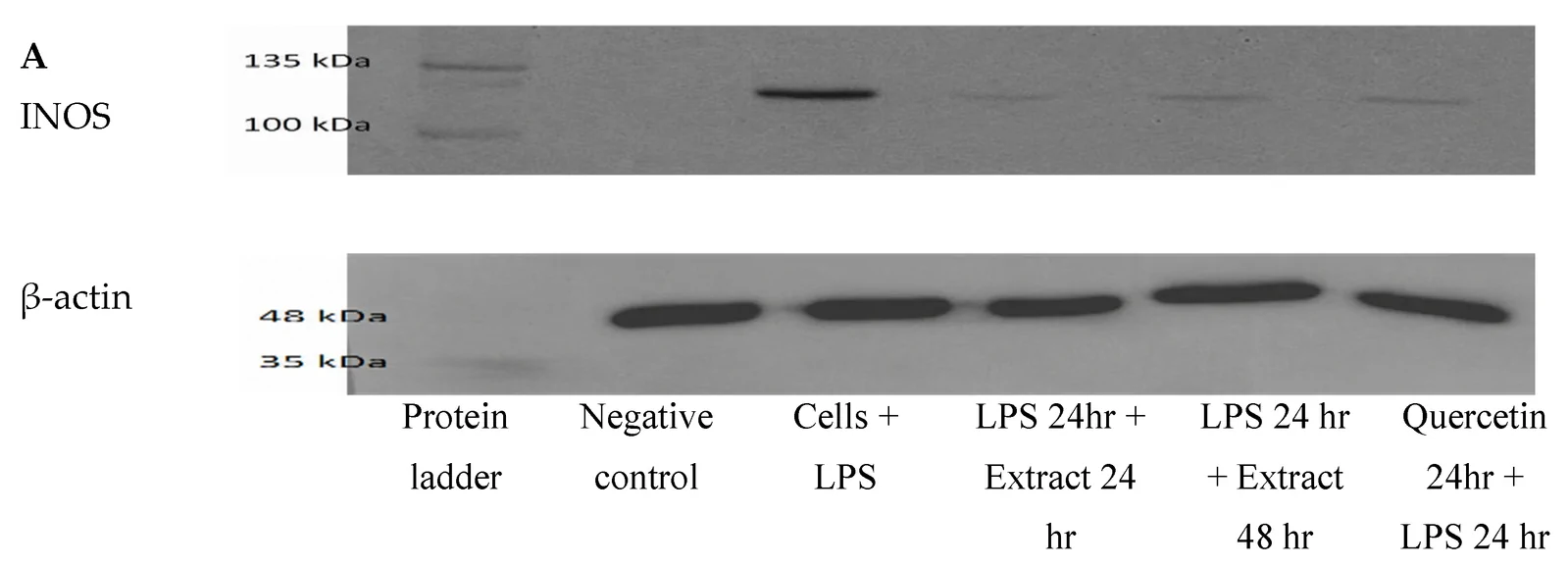

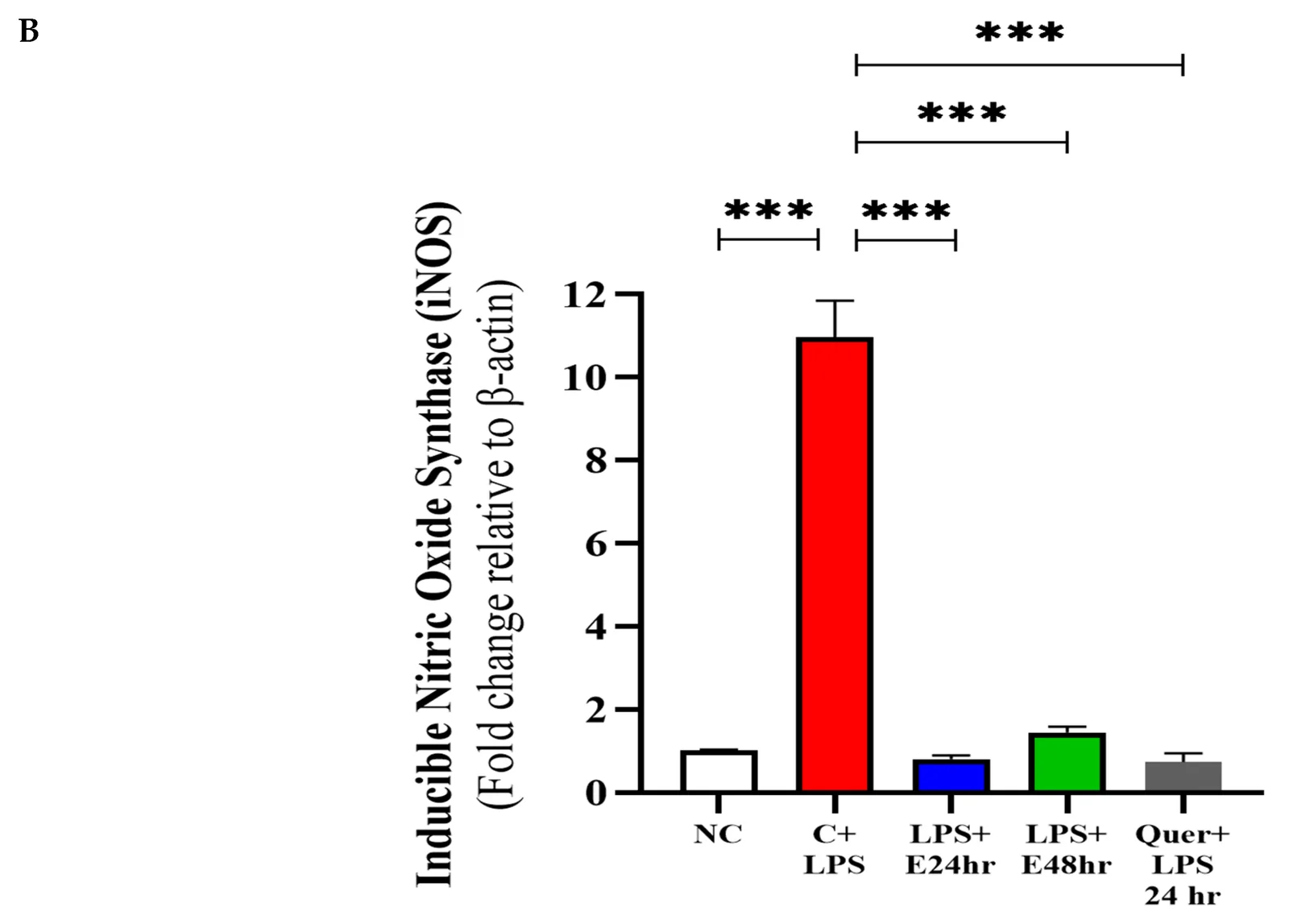
Western blot analysis of iNOS expression in RAW264.7 macrophages following treatment with *T. pinoyi* CCASU-TP-03 extract. (**A**) Representative immunoblot of iNOS and β-actin. (**B**) Relative iNOS protein expression determined by densitometric analysis and normalized to β-actin. C+LPS: Cells and LPS; LPS+E24 h: LPS and Extract for 24 h; LPS+E48 h: LPS and Extract for 48 h; Quer+LPS24 h: Quercetin and LPS for 24 h. Data are expressed as mean ± SD (*n* = 3). Statistical significance (***) was determined relative to the LPS-treated group (*p* < 0.01).

To perform a more thorough investigation into the mechanism that underlies the anti-inflammatory effects of *T. pinoyi*, the expression of iNOS; NOS2 was assessed through Western blot analysis (Figure 3). LPS stimulation significantly elevated the expression of iNOS protein in comparison to the untreated control. Treatment with *T. pinoyi* CCASU-TP-03 extract for 24 h markedly suppressed iNOS expression, reducing it to a level comparable to that of the untreated control. Although a slight increase in iNOS expression was observed after 48 h of treatment compared with the 24 h treatment, the expression level stayed much lower than that of the LPS-treated group. These findings suggest that inhibition of nitric oxide production by the *T. pinoyi* CCASU-TP-03 extract is associated with downregulation of iNOS protein expression. Figure S2 displays a replicate of Western blot analysis of iNOS and β-actin.

### 2.8. Cytotoxic Activity

**Figure 4:**
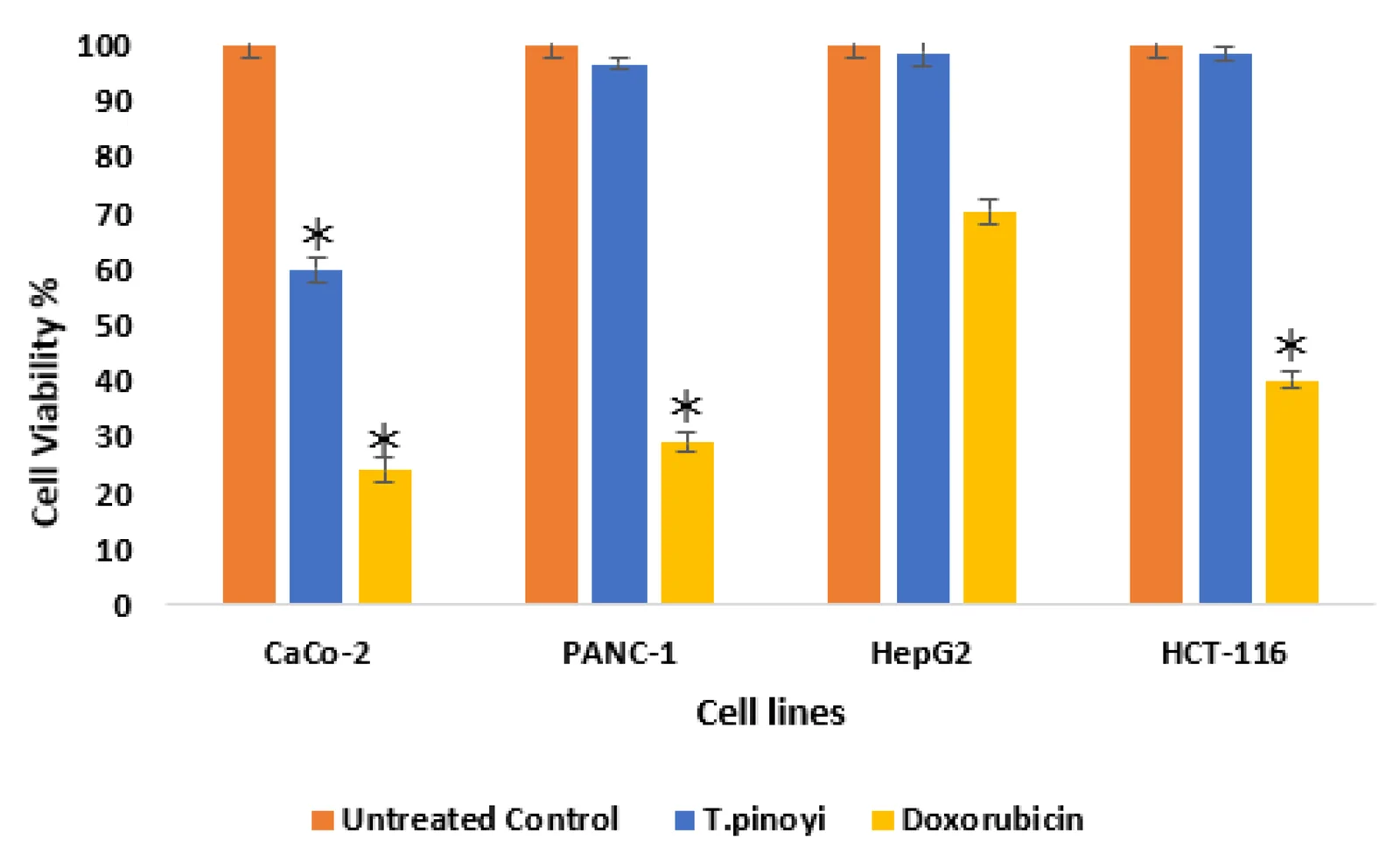
Effect of *T. pinoyi* CCASU-TP-03 extract (100 μg/mL) on the viability of CaCo-2, PANC-1, HepG2, and HCT-116 cells as determined by the sulforhodamine B (SRB) assay. Untreated cells served as the control, while doxorubicin was used as the positive cytotoxic control. Cell viability was expressed relative to the corresponding untreated control cells. Data are presented as mean ± SD. Statistical significance was determined relative to the corresponding untreated control group (*p* < 0.05). * = significant effect.

The cytotoxic effects of *T. pinoyi* CCASU-TP-03 extract were evaluated using the SRB assay over a concentration range of 0.01–100 μg/mL. Among the investigated cell lines, CaCo-2 cells showed the greatest concentration-related reduction in viability, particularly at the higher extract concentrations. In contrast, PANC-1, HepG2, and HCT-116 cells maintained comparatively high viability throughout the tested concentration range. At the highest tested concentration (100 μg/mL), this differential response was also evident in Figure 4. Since 50% inhibition was not reached within the investigated concentration range, the IC_50_ values were considered >100 μg/mL under the present experimental conditions. Doxorubicin was included as the positive cytotoxic control. The complete concentration–response curves for the tested extract concentrations are provided in Figure S3. These findings demonstrate differential effects of the extract on the viability of the tested cancer cell lines under the applied experimental conditions, with CaCo-2 cells showing the greatest reduction in viability among the tested cancer cell lines.

## 3. Discussion

Mushrooms have been prized for their distinctive aroma and excellent nutritional profile, serving dual roles in culinary traditions and folk medicine. Although they are low in calories and fat, they offer a significant supply of proteins, vitamins, minerals, and dietary fiber. This study offers a thorough assessment of the chemical makeup and biological activities of *T. pinoyi* CCASU-TP-03 extract. Phylogenetic placement of CCASU-TP-03 together with reference *T. pinoyi* sequences provides molecular support for the taxonomic identity of the biological material used in the subsequent chemical and in vitro analyses. The chemical characterization of *T. pinoyi* CCASU-TP-03 extract demonstrated measurable total carbohydrate content expressed as glucose equivalents, together with protein, total phenolic, and total flavonoid contents, highlighting the dual nutritional and chemical value of this edible desert truffle. Carbohydrates are recognized as the predominant macronutrients in desert truffles, while proteins contribute substantially to their nutritional quality [15]. The present findings are consistent with previous reports on desert truffles. For example, *Tirmania nivea* collected from Tunisia was reported to contain carbohydrates as the major nutritional component (57.83 g/100 g dry weight), followed by proteins (28.81 g/100 g dry weight), together with appreciable amounts of flavonoids and phenolic compounds, emphasizing the nutritional and functional importance of the genus [16]. The detected phenolic compounds may provide a plausible chemical context for the observed activities; however, their individual contributions cannot be established without fractionation and direct testing of isolated compounds. Likewise, a comparative study of *Tirmania nivea* and *Terfezia boudieri* demonstrated considerable levels of phenolics and flavonoids, although their concentrations varied according to species, sample preparation, and extraction procedure [17].

Targeted HPLC-PDA profiling of *T. pinoyi* CCASU-TP-03 extract indicated the presence of selected phenolic and flavonoid constituents, including gallic acid, ellagic acid, quercetin, and rutin, based on comparison of their retention times and PDA spectral characteristics with authentic reference standards. In contrast, catechin, chlorogenic acid, caffeic acid, hesperidin, kaempferol, and apigenin were not detected under the applied chromatographic conditions. Similar phenolic constituents have been reported in other edible desert truffles, although their profiles may vary considerably among species and according to extraction and analytical conditions [16,17]. Gallic acid, quercetin, ellagic acid, and rutin have each been widely investigated for their antioxidant properties and have also been associated with antimicrobial, anti-inflammatory, and anticancer activities in numerous experimental studies [18,19]. Targeted HPLC-PDA tentatively detected gallic acid, ellagic acid, isoferulic acid, quercetin, and rutin based on comparison of chromatographic retention and PDA spectral characteristics with reference standards. Their detection provides compositional information only, and the activity of the crude extract cannot be causally assigned to any individual constituent. The detection of these metabolites provides a plausible chemical basis for the biological activities exhibited by *T. pinoyi* CCASU-TP-03 extract.

The antioxidant activity identified in both DPPH and ORAC assays is probably linked to the presence of phenolic compounds and flavonoids found in the extract. To counteract free radicals, phenolic compounds can donate electrons or hydrogen atoms, while also chelating transition metals involved in reactive oxygen species generation [20]. The relatively high DPPH IC_50_ observed in the present study suggests limited radical-scavenging potency compared with extracts exhibiting lower IC_50_ values. Differences in extraction procedures, extract concentration, phenolic composition, and assay conditions may contribute to variations in antioxidant activity among truffle species. In comparison to other solvent extracts, the aqueous extract of *T. claveryi* showed a significantly greater total phenolic content (176.67 mg GAE/g DW), and this value was linked with antioxidant activity [21]. According to other research, phenolic compounds are known to stabilize free radicals (DPPH radicals and ABTS radical cations) and decrease metals (FRAP/CUPRAC), creating a barrier against oxidative stress. The findings indicated that Basidiomycota samples exhibit a greater capacity for free radical scavenging in comparison to the examined Ascomycota. Members [20,22]. Several studies have demonstrated a close relationship between phenolic compounds in mushrooms and antioxidant activity across several assays, which is consistent with the current findings [18].

The antimicrobial activity observed in the present study was relatively limited, as indicated by the comparatively high MIC values. The lack of activity against *Aspergillus niger* may indicate lower susceptibility of filamentous fungi or insufficient concentrations of active metabolites against this organism. The antifungal activity observed against *Candida albicans* suggests that *T. pinoyi* CCASU-TP-03 contains compounds capable of interfering with fungal growth. The enhanced efficacy of methanol extracts aligns with earlier findings, which attribute the primary antimicrobial activity against fungi to polar-solvent-extracted biochemicals, including phenolic compounds, flavonoids, and organic acids [23,24]. Truffles and other macrofungi are notably abundant in phenolic acids and bioactive peptides, which can interfere with vital metabolic processes and damage bacterial cell membranes to produce antibiotic actions [25]. The greater susceptibility of the tested Gram-positive bacteria compared with the Gram-negative bacterium may be related to differences in cell-envelope structure, although the present study did not investigate the underlying mechanism. Differences from previously reported antimicrobial activities of desert truffles may also be attributable to variations in fungal species, extraction solvents, extract concentrations, and test organisms.

The extract demonstrated differential effects on the viability of the tested cancer cell lines. Concentration–response analysis showed that CaCo-2 cells were the most responsive to the extract, whereas PANC-1, HepG2, and HCT-116 cells maintained comparatively high viability throughout the tested concentration range of 0.01–100 μg/mL. Since 50% inhibition was not reached within this range, the IC_50_ values were considered >100 μg/mL under the present experimental conditions. These findings indicate comparatively greater sensitivity of CaCo-2 cells, although the observed effect should be regarded as preliminary and does not establish anticancer selectivity. Previous studies have reported moderate cytotoxic activity of methanolic extracts of *T. nivea* and *T. claveryi* against HepG2 and L929 cells, although IC_50_ values could not be determined [26]. Differences in sensitivity among cell lines may be related to variations in cellular metabolism, membrane permeability, genetic background, and intracellular signaling pathways [27]. Although the precise mechanism responsible for the observed cytotoxicity remains to be elucidated, secondary metabolites identified in the extract have previously been associated with modulation of oxidative stress, cell cycle progression, and apoptosis [28]. The present findings do not establish a causal relationship between these constituents and the observed cytotoxicity. Furthermore, specific intracellular signaling pathways, apoptosis markers, cell-cycle progression, and molecular targets were not investigated in the present study. Therefore, the observed cytotoxic activity, particularly the greater sensitivity of CaCo-2 cells, should be regarded as preliminary evidence warranting further mechanistic and selectivity studies [29,30,31,32,33].

One important component Inflammation is an important component of many chronic diseases and is characterized by increased production of inflammatory mediators, including nitric oxide (NO). In the present study, *T. pinoyi* CCASU-TP-03 extract reduced LPS-induced NO production, with the most pronounced inhibition observed at 500 μg/mL (40.23%). Importantly, RAW264.7 cell viability remained above 97% throughout the investigated concentration range (31.25–500 μg/mL), including a viability of 97.50% at 500 μg/mL, indicating that the observed reduction in NO production was unlikely to be attributable to an appreciable loss of cell viability. The marked increase in NO inhibition from 5.42% at 250 μg/mL to 40.23% at 500 μg/mL suggests a nonlinear, threshold-like response of the crude extract at higher concentrations rather than a gradual concentration-dependent effect; however, because intermediate concentrations between 250 and 500 μg/mL were not evaluated, the precise concentration–response relationship cannot be defined. The greater reduction in iNOS protein expression compared with the approximately 40% inhibition of NO production may reflect the different nature and timing of the two measurements. Western blotting assesses iNOS protein abundance at a defined sampling time, whereas the Griess assay measures nitrite accumulated in the culture medium during the incubation period. Therefore, changes in iNOS protein expression and accumulated NO production are not necessarily expected to occur to the same magnitude.

Western blot analysis further demonstrated reduced iNOS protein expression following treatment with the extract. Since iNOS is responsible for high-output NO production during inflammatory stimulation, the parallel reduction in NO production and iNOS expression, together with preservation of RAW264.7 cell viability, supports an inhibitory effect of the extract on the iNOS/NO inflammatory response under the present experimental conditions. A previous study reported 21.7% NO inhibition by *T. pinoyi* extract [34]. However, direct comparison with the present value should be interpreted cautiously because differences in extract preparation, concentration, and experimental conditions may influence the magnitude of the observed response. Collectively, these findings suggest that modulation of the iNOS/NO pathway may contribute to the anti-inflammatory activity of *T. pinoyi* CCASU-TP-03 extract. The present findings indicate that *T. pinoyi* CCASU-TP-03 is a valuable source of naturally occurring bioactive compounds with diverse biological activities. These findings imply that *T. pinoyi* CCASU-TP-03 could be a viable natural source of physiologically active substances with potential uses in nutraceuticals and functional foods.

## 4. Materials and Methods

### 4.1. Chemicals and Reagents

The principal chemicals and reagents used in this study included methanol (El Gomhouria Co., Cairo, Egypt), distilled water, Folin–Ciocalteu reagent, sodium carbonate (Na_2_CO_3_), aluminum chloride, gallic acid, rutin, phosphoric acid, and bicinchoninic acid (BCA) assay reagents (Sigma-Aldrich, St. Louis, MO, USA) for chemical characterization of the extract. Methanol and 0.1% phosphoric acid in water were used as the mobile phases for HPLC-PDA analysis, together with the corresponding phenolic and flavonoid reference standards (Merck, Darmstadt, Germany). Dimethyl sulfoxide (DMSO) (Sigma-Aldrich, St. Louis, MO, USA) was used as a solubilizing solvent in the antimicrobial assay, while gentamicin and ketoconazole (Thermo Fisher Scientific, Waltham, MA, USA) were used as positive antibacterial and antifungal controls, respectively. The appropriate broth medium was used for preparation of the antimicrobial assay. For the antioxidant assays, 2,2-diphenyl-1-picrylhydrazyl (DPPH), Trolox, fluorescein, and 2,2′-azobis(2-amidinopropane) dihydrochloride (AAPH) (Sigma-Aldrich, St. Louis, MO, USA) were used. For the anti-inflammatory experiments, lipopolysaccharide (LPS) (Bio-Rad Laboratories, Hercules, CA, USA) was used to stimulate RAW264.7 macrophages, dexamethasone (Thermo Fisher Scientific, Waltham, MA, USA))served as the positive control in the nitric oxide assay, and quercetin (Sigma-Aldrich, St. Louis, MO, USA) was used as the reference treatment in the Western blot experiment. For the cytotoxicity assay, complete growth medium, sulforhodamine B (SRB), trichloroacetic acid (TCA), acetic acid, and Tris base (Gibco, Grand Island, NY, USA) were used, while doxorubicin (Thermo Fisher Scientific, Waltham, MA, USA) served as the positive cytotoxic control.

### 4.2. Collection and Identification of Truffle Isolate

Truffle samples were collected from the deserts of Jizan City, Saudi Arabia. The collected specimens were morphologically and molecularly identified using partial 25S large-subunit ribosomal DNA and the internal transcribed spacer region comprising ITS1, 5.8S rRNA, and ITS2, as previously reported [35]. Total genomic DNA was extracted using Zymo Research (ZR) Fungal/Bacterial DNA kit™ (Zymo Research, Irvine, CA, USA) following the manufacturer’s instructions. The used primers and PCR conditions were carried out as previously described [6,36]. The phylogenetic trees of both the large subunit and ITS1–5.8S rRNA–ITS2 region were constructed for the consensus-aligned sequences (MUSCLE alignment) using the maximum likelihood method in MEGA12, as previously reported [37]. The reference parameters of the phylogenetic analysis included the Tamura-Nei substitution model, standard Bootstrap as a test of phylogeny, with 1000 replicates. The rates and pattern were uniform, and the number of threads was 4. Three specimens were used for DNA sequencing in both forward and reverse directions, and the consensus sequences were obtained using the Staden package software version 2. The final consensus sequences of both small and large ribosomal subunits have been submitted to the NCBI GenBank database, and the accession codes were obtained.

### 4.3. Extract Preparation

Air-dried fruiting bodies of *T. pinoyi* CCASU-TP-03 (250 g) were ground into a fine powder and macerated in 70% methanol at a sample-to-solvent ratio of 1:30, *w*/*v* for two weeks at 25 °C, with periodic agitation. The extract was filtered through Whatman No. 4 filter paper, and the solvent was removed under reduced pressure at 40 °C using a rotary evaporator. Because the stability of individual metabolites was not monitored during the prolonged maceration period, the reported chemical profile should be interpreted as the composition of the recovered crude extract rather than as an unchanged representation of the native fruiting-body metabolome. Approximately 69.8 g of dry crude extract was obtained. The dried extract was stored at 4 °C in clean, tightly closed containers until analysis [38].

### 4.4. Chemical Characterization of the Mushroom Extract

#### 4.4.1. Total Content of Carbohydrates

The total carbohydrate content was measured using a colorimetric technique, as described by Yue et al. [39].; measurements were obtained from three independent experimental determinations (*n* = 3).

#### 4.4.2. Total Flavonoid and Phenolic Content

Total phenolic content (TPC) and total flavonoid content (TFC) were determined using the Folin–Ciocalteu and aluminum chloride colorimetric methods, respectively [40,41]. Samples were prepared at a 20 mg/mL concentration in distilled water. Briefly, the procedure of TPC consisted of mixing 10 μL of sample/standard with 100 μL of Folin–Ciocalteu reagent (Diluted 1: 10) in a 96-well microplate. Then, 80 μL of 1 M Na_2_CO_3_ was added and incubated at room temperature (25 °C) for 20 min in the dark. Stock solutions of gallic acid and rutin (1 mg/mL) were created in methanol and subsequently serially diluted to establish calibration curves. At the end of the incubation time, the resulting blue complex color was measured at 630 nm. For TFC, Briefly, 15 μL of sample/standard was placed in a 96-well microplate, then 175 μL of methanol was added, followed by 30 μL of 1.25% AlCl3. Finally, 30 μL of 0.125 M C_2_H_3_NaO_2_ was added and incubated for 5 min. At the end of incubation time the resulting yellow color was measured at 420 nm. Gallic acid equivalents (GAE) and rutin equivalents (RE) were used to express the results of all assays, which were carried out in triplicate.

#### 4.4.3. Targeted HPLC-PDA Analysis of Selected Phenolic and Flavonoid Compounds

HPLC-PDA analysis was performed using a Waters 2690 Alliance HPLC system (Waters Corporation, Milford, MA, USA) equipped with a Waters 996 photodiode array detector (Waters Corporation, Milford, MA, USA). Separation was achieved using an Inertsil ODS-3 C18 column (4.6 × 250 mm, 5 μm particle size) maintained at ambient temperature. The mobile phase consisted of 0.1% orthophosphoric acid in water (solvent A) and methanol (solvent B) at a flow rate of 1.0 mL/min. Gradient elution was programmed as follows: 0–3 min, 5% B; 3–50 min, 5–50% B; 50–55 min, 50–70% B; 55–75 min, 70–90% B; 75–76 min, 90–5% B; and 76–80 min, 5% B. The total run time was 80 min, including a 4-min re-equilibration period at the initial mobile-phase composition. The injection volume was 10 μL, and chromatographic detection was performed at 280 nm, their UV spectra and retention times were compared to those of valid standards.

#### 4.4.4. Total Protein Content

Total protein content was determined using the bicinchoninic acid (BCA) assay as previously reported; measurements were obtained from three independent experimental determinations (*n* = 3) [42].

### 4.5. Antimicrobial Activity Assay

The antimicrobial activity of *T. pinoyi* extract against *S. aureus* ATCC 25923, *B. subtilis* RCMB 015 (1) NRRL B-543, *E. coli* ATCC 25922, *P. vulgaris* RCMB 004 (1) ATCC 13315, *C. albicans* ATCC 10231, and *A. niger* RCMB 002005 was evaluated using the broth microdilution method according to CLSI guidelines [43]. A concentrated stock solution of the extract was prepared using DMSO as the solubilizing solvent and subsequently diluted with the appropriate broth medium, while maintaining the final DMSO concentration at ≤1% (*v*/*v*) in all assay wells. Two-fold serial dilutions of the stock solution were prepared, and after mixing equal volumes of the extract and microbial inoculum, the final extract concentrations in the wells ranged from 25 to 0.391 mg/mL. Microbial suspensions (1 × 10^6^ CFU/mL, 0.5 McFarland) were mixed with equal volumes of each extract dilution in 96-well plates and incubated at 35 ± 1 °C for 24 h, except *C. albicans* and *A. niger*, which were incubated at 22.5 ± 2.5 °C for 48 h. Following incubation, microbial growth was assessed visually by comparison with the corresponding growth-control wells, and the MIC was defined as the lowest concentration of the extract showing no visible microbial growth. Gentamicin (4 μg/mL) and ketoconazole (10 μg/mL) were used as positive controls. The experiment was performed in three independent replicates.

### 4.6. Evaluation of Antioxidant Properties

#### 4.6.1. DPPH Radical Scavenging Activity

The antioxidant activity of *T. pinoyi* extract was evaluated using the DPPH radical scavenging assay according to Salem et al. [44]. A stock solution of the extract (10 mg/mL) was prepared and diluted to final concentrations of 250–2000 μg/mL, while Trolox served as the reference antioxidant. Briefly, 100 μL of freshly prepared DPPH solution (0.1% in methanol) was mixed with 100 μL of each extract concentration in 96-well microplates, and the mixtures were incubated in the dark at room temperature for 30 min. Absorbance was measured at 540 nm using a FluoStar Omega microplate reader (V6.40). The percentage inhibition of DPPH radicals was calculated according to Equation (1), and the half-maximal inhibitory concentration (IC_50_) was determined by nonlinear regression analysis using GraphPad Prism 6 software. Results are expressed as mean ± standard deviation (SD).

(1) $$\mathrm{Percentage inhibition}\%=\frac{\left(\mathrm{Average absorbance of blank}-\mathrm{Average absorbance of the extract}\right)}{\mathrm{Average absorbance of blank}}\times 100$$

#### 4.6.2. ORAC Assay

The ORAC of *T. pinoyi* extract was measured as previously described [45]. The extract (1 mg/mL) was mixed with fluorescein (100 nM) in black 96-well plates and incubated at 37 °C for 10 min. After recording the initial fluorescence (Ex 485 nm, Em 520 nm), AAPH (240 mM) was added, and fluorescence was monitored for 60 min. Antioxidant capacity was expressed as μM Trolox equivalents (μM TE/mg sample) using a Trolox standard curve.

### 4.7. Evaluation of Anti-Inflammatory

The anti-inflammatory activity of *T. pinoyi* extract was determined by measuring nitric oxide (NO) production in LPS-stimulated RAW 264.7 murine macrophages obtained from Nawah Scientific Inc. (Cairo, Egypt). Cells were cultured in DMEM supplemented with 10% FBS, 100 U/mL penicillin, and 100 μg/mL streptomycin at 37 °C in a humidified 5% CO_2_ atmosphere. RAW 264.7 cells were seeded into 96-well plates and incubated for 24 h before stimulation with LPS (1 μg/mL) in the presence or absence of different concentrations of *T. pinoyi* extract. Dexamethasone (1 μM) served as the positive control. Nitric oxide production was determined by the Griess assay, and absorbance was measured at 540 nm using a microplate reader. Anti-inflammatory activity was expressed as the percentage inhibition of NO production relative to the LPS-treated control [14].

### 4.8. Western Blot Analysis

To investigate the effect of *T. pinoyi* extract on iNOS expression, RAW264.7 macrophages were divided into the following treatment groups: untreated control cells; cells stimulated with LPS for 24 h; cells stimulated with LPS for 24 h followed by treatment with *T. pinoyi* CCASU-TP-03 extract (500 μg/mL) for 24 h; cells stimulated with LPS for 24 h followed by extract treatment (500 μg/mL) for 48 h; and cells treated with quercetin (30 µM) in the presence of LPS for 24 h as a positive control. Following the respective treatment periods, iNOS protein expression was evaluated by Western blotting. β-Actin was used as the loading control, and iNOS expression was normalized to β-actin [46].

### 4.9. Cytotoxic Activity

The cytotoxic activity of *T. pinoyi* extract was evaluated against HCT-116, CaCo-2, HepG2, and PANC-1 cancer cell lines. All cell lines were obtained from Nawah Scientific Inc. (Cairo, Egypt). Cell viability was determined using the sulforhodamine B (SRB) assay. Briefly, cells were seeded into 96-well plates at a density of 5 × 10^3^ cells/well and incubated in complete growth medium for 24 h. Subsequently, cells were treated with various concentrations of *T. pinoyi* extract ranging from 0.01 to 100 μg/mL and incubated for 72 h under standard culture conditions. Following the exposure period, cells were fixed with 10% trichloroacetic acid (TCA) at 4 °C for 1 h and washed thoroughly with distilled water. Cells were then stained with 0.4% (*w*/*v*) sulforhodamine B solution for 10 min at room temperature in the dark. Excess dye was removed by washing with 1% acetic acid, and the plates were allowed to air-dry overnight. Protein-bound dye was dissolved in 10 mM Tris base, and absorbance was measured at 540 nm using an Infinite F50 microplate reader (TECAN, Männedorf, Switzerland). Cell viability was expressed as a percentage relative to untreated control cellsCell viability was expressed as a percentage relative to untreated controls. Doxorubicin (100 μg/mL) was used as the positive control [47].

### 4.10. Statistical Analysis

The data were represented using the mean ± standard deviation. A one-way analysis of variance (ANOVA) was conducted for statistical evaluation. The Tukey–Kramer multiple comparison tests were employed to determine differences between groups. Differences were considered statistically significant at *p* < 0.05.

## 5. Conclusions

The present study provides a chemical and in vitro biological characterization of the edible desert truffle *Tirmania pinoyi* CCASU-TP-03. All biological effects reported in the present study were obtained using the crude 70% methanolic extract of *T. pinoyi* CCASU-TP-03 and should not be attributed to any individual compound identified by targeted HPLC-PDA. The extract contained measurable levels of carbohydrates, total phenolics, total flavonoids, and protein, while targeted HPLC-PDA analysis detected several selected phenolic and flavonoid compounds, including gallic acid, ellagic acid, isoferulic acid, quercetin, and rutin. The extract exhibited measurable antioxidant capacity, inhibitory activity against several tested microorganisms, differential effects on the viability of the tested cancer cell lines, and inhibition of NO production in LPS-stimulated RAW264.7 macrophages accompanied by reduced iNOS protein expression. The relatively high antimicrobial MIC values and DPPH IC_50_, together with the targeted nature of the HPLC-PDA analysis and the absence of comprehensive metabolomic and mechanistic investigations, should be considered when interpreting these findings. Further studies employing comprehensive chemical profiling, concentration-response analyses, and mechanistic investigations are warranted to identify the constituents that may contribute to the observed biological effects.

## Figures and Tables

**Table 1:** Chemical characterization of the *T. pinoyi* CCASU-TP-03 extract.

Mushroom	Total Carbohydrate Content (mg G eq/g Extract)	Total Phenolic Content (mg GAE/g Extract)	Total Flavonoid Content (mg RE/g Extract)	Total Protein Content (mg/g Extract)
*T. pinoyi*	354.245 ± 7.705	72.572 ± 0.119	9.831 ± 0.023	11.12 ± 0.57

All outcomes are presented as mean ± SD derived from three separate experiments (*n* = 3).

**Table 2:** Targeted qualitative HPLC-PDA profiling of selected phenolic and flavonoid standards in *Tirmania pinoyi* CCASU-TP-03 extract based on comparison of retention times and PDA spectral characteristics with authentic reference standards.

Compounds	Retention Time (min)	Result
Gallic acid	10.35	Detected
Catechin	24.58	ND *
Isoferulic acid	28.20	Detected
Chlorogenic acid	28.65	ND *
Caffeic acid	29.37	ND *
Hesperidin	45.34	ND *
Rutin	47.60	Detected
Ellagic acid	49.39	Detected
Quercetin	55.50	Detected
Kaempferol	58.16	ND *
Apigenin	58.69	ND *

ND * = Not detected under the applied chromatographic conditions.

**Table 3:** The MICs of *T. pinoyi* CCASU-TP-03 extract. Values represent the results obtained from three independent experiments.

Microbial Strains	MIC (mg/mL)
*S. aureus* ATCC 25923	12.5
*B. subtilis* RCMB 015 (1) NRRL B-543	3.125
*E. coli* ATCC 25922	25
*P. vulgaris* RCMB 004 (1) ATCC 13315	12.5
*C. albicans* ATCC 10231	6.25
*A. niger* RCMB 002005	>25

Minimum inhibitory concentrations (MICs) of *T. pinoyi* extract against the tested bacterial and yeast strains. MIC > 25 mg/mL indicates that complete inhibition of visible growth was not observed at the highest extract concentration tested.

**Table 4:** Antioxidant activity of *T. pinoyi* CCASU-TP-03 extract determined by DPPH and ORAC assays.

Assay	*T. pinoyi* CCASU-TP-03
DPPH radical scavenging activity (IC_50_, μg/mL)	1070 ± 1.03
ORAC (μmol TE/mg extract)	231.12 ± 26.86

Values are expressed as mean ± SD (*n* = 3). IC_50_: half-maximal inhibitory concentration; TE: Trolox equivalents [14].

**Table 5:** Inhibitory effect of *T. pinoyi* CCASU-TP-03 extract on nitric oxide production in LPS-stimulated RAW 264.7 macrophages. Values are expressed as mean ± SD of three independent experiments. Different superscript letters indicate statistically significant differences between groups (*p* < 0.05).

*T. pinoyi* CCASU-TP-03 (μg/mL)	NO Inhibition (%) ± SD
31.25	0.94 ± 0.21 ^c^
62.5	0.74 ± 0.00 ^c^
125	3.90 ± 0.59 ^b^
250	5.42 ± 0.44 ^b^
500	40.23 ± 1.82 ^a^

## Data Availability

The original contributions presented in this study are included in the article. The 25S rRNA gene sequence of *T. pinoyi* CCASU-TP-03 is available in the NCBI GenBank database under accession number PZ697207 (https://www.ncbi.nlm.nih.gov/nuccore/PZ697207.1) (accessed on 11 October 2026). The ITS1–5.8S rRNA–ITS2 region (internal transcribed spacer 1 and 5.8S ribosomal RNA gene, and internal transcribed spacer 2; (NCBI accession: PZ953577 (https://www.ncbi.nlm.nih.gov/nuccore/PZ953577.1) (accessed on 11 October 2026). All other data are included in the published article.

## Supplementary Materials

The following supporting information can be downloaded at https://www.mdpi.com/article/10.3390/ijms27219925/s1.

## References

[1] Heleno S.A., Barros L., Martins A., Morales P., Fernández-Ruiz V., Glamoclija J., Sokovic M., Ferreira I.C.F.R. (2015). Nutritional Value, Bioactive Compounds, Antimicrobial Activity and Bioaccessibility Studies with Wild Edible Mushrooms. LWT-Food Sci. Technol..

[2] Aprotosoaie A.C., Zavastin D.E., Mihai C.-T., Voichita G., Gherghel D., Silion M., Trifan A., Miron A. (2017). Antioxidant and Antigenotoxic Potential of *Ramaria largentii* Marr & D. E. Stuntz, a Wild Edible Mushroom Collected from Northeast Romania. Food Chem. Toxicol..

[3] Altaf U., Lalotra P., Sharma Y.P. (2020). Nutritional and Mineral Composition of Four Wild Edible Mushrooms from Jammu and Kashmir, India. Indian Phytopathol..

[4] Sridhar K., Deshmukh S. (2025). Truffles.

[5] Tejedor-Calvo E., Amara K., Reis F.S., Barros L., Martins A., Calhelha R.C., Venturini M.E., Blanco D., Redondo D., Marco P. (2021). Chemical Composition and Evaluation of Antioxidant, Antimicrobial and Antiproliferative Activities of Tuber and Terfezia Truffles. Food Res. Int..

[6] Elhusseiny S.M., El-Mahdy T.S., Awad M.F., Elleboudy N.S., Farag M.M.S., Aboshanab K.M., Yassien M.A. (2021). Antiviral, Cytotoxic, and Antioxidant Activities of Three Edible Agaricomycetes Mushrooms: *Pleurotus columbinus*, *Pleurotus sajor-caju*, and *Agaricus bisporus*. J. Fungi.

[7] Tellez-Tellez M. (2024). Wild Edible Mushrooms as an Alternative for the Consumption of Antioxidants and Phenolic Compounds: An Overview. Bioresources.

[8] Liu K., Xiao X., Wang J., Chen C.-Y.O., Hu H. (2017). Polyphenolic Composition and Antioxidant, Antiproliferative, and Antimicrobial Activities of Mushroom *Inonotus sanghuang*. LWT-Food Sci. Technol..

[9] Yan H., Che X., Lv Q., Zhang L., Dongol S., Wang Y., Sun H., Jiang J. (2017). Grifolin Induces Apoptosis and Promotes Cell Cycle Arrest in the A2780 Human Ovarian Cancer Cell Line via Inactivation of the ERK1/2 and Akt Pathways. Oncol. Lett..

[10] Macharia J.M., Zhang L., Mwangi R.W., Rozmann N., Kaposztas Z., Varjas T., Sugár M., Alfatafta H., Pintér M., Bence R.L. (2022). Are Chemical Compounds in Medical Mushrooms Potent against Colorectal Cancer Carcinogenesis and Antimicrobial Growth?. Cancer Cell Int..

[11] Neggaz S., Korinek M., El-Shazly M., Bensouici C., El Haouaria Zitouni-Haouar F., Gherram M., Hwang T.-L., Fernandez X. (2026). First Assessment of the Bioactive Content of the Edible Desert Truffle Tirmania Sahariensis with Acetylcholinesterase Inhibitory, Antioxidant, Antimicrobial, Anti-Allergic and Anti-Inflammatory Properties. J. Food Meas. Charact..

[12] Qurie M., Khader A., AbuKadum A., Salah Z., Jaffal A., Shkhadm N., Salemeh S. (2026). Bioactive Properties of Terfezia Arenaria Extract: Antioxidant, Antimicrobial, Anticancer, and Enzyme Inhibition Effects. Arab. J. Sci. Eng..

[13] Ghanney N., Ben Yahia L., Hlel H., Lachiheb B., Bouhamada T., Fakhfakh N., Nagaz K. (2026). Impact of Seven Conservation Methods on the Nutritional Value, Phytochemical Composition, and Chemical Antioxidant Capacity of *Terfezia boudieri* and *Tirmania nivea* from Southeastern Tunisia. Front. Pharmacol..

[14] Joo T., Sowndhararajan K., Hong S., Lee J., Park S.-Y., Kim S., Jhoo J.-W. (2014). Inhibition of Nitric Oxide Production in LPS-Stimulated RAW 264.7 Cells by Stem Bark of *Ulmus pumila* L. Saudi J. Biol. Sci..

[15] Aljamal E., Ragab I.I., Ashour A., Melhem M.B. (2026). Analytical Standardization and Bioactivity Assessment of Desert Truffles (*Terfezia* Spp.): A Critical Mini-Review. J. Food Meas. Charact..

[16] Hamza A., Jdir H. (2016). Nutritional, Antioxidant and Antibacterial Properties of *Tirmania nivea*, A Wild Edible Desert Truffle from Tunisia Arid Zone. Med. Aromat. Plants.

[17] Hammami R., Alsaloom A.N., Aouadhi C., Alrokban A., Mendili M., Khadhri A. (2023). HPLC Analysis, Mycochemical Contents and Biological Activities of Two Edible Hypogeous Ascomycetes: *Tirmania nivea* and *Terfezia boudieri*. Heliyon.

[18] Ildız E., Yürümez Canpolat E. (2026). Phenolic-Rich Wild Edible Macrofungi: Antimicrobial Activity and Antioxidant Potential. Molecules.

[19] Shakuri F., Eghlima G., Behboudi H., Babashpour-Asl M. (2025). Phytochemical Variation, Phenolic Compounds and Antioxidant Activity of Wild Populations of Iranian Oak. Sci. Rep..

[20] Rao M.J., Zheng B. (2025). The Role of Polyphenols in Abiotic Stress Tolerance and Their Antioxidant Properties to Scavenge Reactive Oxygen Species and Free Radicals. Antioxidants.

[21] Hadjira G., Bakchìche B., Erenler R., Yìldìz İ., Mohamed A., EL-shazly M.A. (2023). Mineral Contents, Antioxidant and Antimicrobial Activities of Algerian *Terfezia claveryi* Extracts. Fabad J. Pharm. Sci..

[22] Saini R., Dhiman N.K. (2021). Natural Anti-Inflammatory and Anti-Allergy Agents: Herbs and Botanical Ingredients. Antiinflamm. Antiallergy Agents Med. Chem..

[23] Heleno S.A., Martins A., Queiroz M.J.R.P., Ferreira I.C.F.R. (2015). Bioactivity of Phenolic Acids: Metabolites versus Parent Compounds: A Review. Food Chem..

[24] Alves M.J., Ferreira I.C.F.R., Froufe H.J.C., Abreu R.M.V., Martins A., Pintado M. (2013). Antimicrobial Activity of Phenolic Compounds Identified in Wild Mushrooms, SAR Analysis and Docking Studies. J. Appl. Microbiol..

[25] Puia A., Pandrea S.-L., Cruceru J., Puia I.C., Chedea V.S., Ciont C., Pop O.L., Leopold L.F., Ranga F., Urcan A.C. (2025). Phenolic Compounds with Antimicrobial Properties in Mushrooms Frequently Encountered in Temperate Deciduous Forests. Life.

[26] Elsayed E., Alsahli F., El Enshasy H., Wadaan M. (2019). Cytotoxic Activities of Different Solvent Extracts of *Tirmania nivea* and *Terfezia claveryi* against HepG2 and L929 Cells. J. Sci. Ind. Res..

[27] Górska-Jakubowska S., Wu Y., Turło J., Xu B. (2025). Critical Review on the Anti-Tumor Activity of Bioactive Compounds from Edible and Medicinal Mushrooms over the Last Five Years. Nutrients.

[28] Sawaya K., Abou Najem S., Khawaja G., Khalil M. (2023). Proapoptotic and Antiproliferative Effects of the Desert Truffle *Terfezia boudieri* on Colon Cancer Cell Lines. Evid. Based Complement. Altern. Med..

[29] Gao H., Shi D., Yin C., Fan X., Cheng X., Qiao X., Liu C., Hu G., Yao F., Qiu J. (2024). A Highly Branched Glucomannan from the Fruiting Body of *Schizophyllum commune*: Structural Characteristics and Antitumor Properties Analysis. Int. J. Biol. Macromol..

[30] Jian L., He Z., Liu S. (2022). Polysaccharide Peptide Induced Colorectal Cancer Cells Apoptosis by Down-Regulating EGFR and PD-L1 Expression. Iran. J. Pharm. Res..

[31] Zhang Y., Zhou R., Liu F., Ng T.B. (2021). Purification and Characterization of a Novel Protein with Activity against Non-Small-Cell Lung Cancer In Vitro and In Vivo from the Edible Mushroom *Boletus edulis*. Int. J. Biol. Macromol..

[32] Larypoor M. (2022). Investigation of HER-3 Gene Expression under the Influence of Carbohydrate Biopolymers Extract of Shiitake and Reishi in MCF-7 Cell Line. Mol. Biol. Rep..

[33] Xu Y., Huang C., Xu T., Xiang X., Amakye W.K., Zhao Z., Yao M., Zhu Y., Ren J. (2024). A Water Polysaccharide–Protein Complex from *Grifola frondosa* Inhibit the Growth of Subcutaneous but Not Peritoneal Colon Tumor under Fasting Condition. Mol. Nutr. Food Res..

[34] Elkhateeb W.A., Soliman A.A.F., Shaheen M.N., Elmahdy E.M., Daba G.M. (2024). Bioactive Potentials of the Truffle Mushrooms *Tirmania nivea*, *Tirmania pinoyi* and *Tuber indicum*. Egypt. Pharm. J..

[35] Zitouni-Haouar F.E.-H., Bidartondo M.I., Moreno G., Carlavilla J.R., Manjón J.L., Neggaz S., Zitouni-Nourine S.H. (2023). Bioclimatic Origin Shapes Phylogenetic Structure of Tirmania (Pezizaceae): New Species and New Record from North Africa. J. Fungi.

[36] Das S.K., Mandal A., Datta A.K., Gupta S., Paul R., Saha A., Sengupta S., Dubey P.K. (2013). Nucleotide Sequencing and Identification of Some Wild Mushrooms. Sci. World J..

[37] Bawono P., Heringa J., Brahme A. (2014). Phylogenetic Analyses. Comprehensive Biomedical Physics.

[38] Nameni A., Bagherzade G., Moudi M., Ghamari Kargar P. (2022). Examination of the Chemical Profile of Methanolic Extract of *Agaricus bisporus* Wild Edible Mushroom, Zarnagh Region (East Azerbaijan Province, Iran). J. Hortic. Postharvest Res..

[39] Yue F., Zhang J., Xu J., Niu T., Lü X., Liu M. (2022). Effects of Monosaccharide Composition on Quantitative Analysis of Total Sugar Content by Phenol-Sulfuric Acid Method. Front. Nutr..

[40] Attard E. (2013). A Rapid Microtitre Plate Folin-Ciocalteu Method for the Assessment of Polyphenols. Open Life Sci..

[41] Sari K.R.P., Ikawati Z., Danarti R., Hertiani T. (2023). Micro-Titer Plate Assay for Measurement of Total Phenolic and Total Flavonoid Contents in Medicinal Plant Extracts. Arab. J. Chem..

[42] Parween S., Nahar P. (2023). Sensitive and Rapid Bradford and BCA Protein Assay by a Common Desktop Scanner. Biomedicine.

[43] CLSI (2025). Performance Standards for Antimicrobial Susceptibility Testing (35th Ed.). CLSI Supplement M100 (35 Ed.). Clinical and Laboratory Standards Institute. https://clsi.org/shop/standards/m100/.

[44] Salem M.A., Mohamed O.G., Mosalam E.M., Elberri A.I., Abdel-Bar H.M., Hassan M., Al-Karmalawy A.A., Tripathi A., Ezzat S.M., Abo Mansour H.E. (2023). Investigation of the Phytochemical Composition, Antioxidant, Antibacterial, Anti-Osteoarthritis, and Wound Healing Activities of Selected Vegetable Waste. Sci. Rep..

[45] Asma U., Bertotti M.L., Zamai S., Arnold M., Amorati R., Scampicchio M. (2024). A Kinetic Approach to Oxygen Radical Absorbance Capacity (ORAC): Restoring Order to the Antioxidant Activity of Hydroxycinnamic Acids and Fruit Juices. Antioxidants.

[46] Kang K.-Y., Park K.-W. (2025). Suppression of LPS-Induced Inflammation by Phragmites Communis Young Leaf Extract via Multi-Target Inhibition of IκB, AP-1, and STAT1/3 Pathways in RAW 264.7 Cells. Plants.

[47] Skehan P., Storeng R., Scudiero D., Monks A., McMahon J., Vistica D., Warren J.T., Bokesch H., Kenney S., Boyd M.R. (1990). New Colorimetric Cytotoxicity Assay for Anticancer-Drug Screening. JNCI J. Natl. Cancer Inst..

