# Supplementary material for: Chemical Characterization and In Vitro Biological Activity of Edible Desert Truffle Tirmania pinoyi CCASU-TP-03
Source: Int J Mol Sci. 2026 Nov 5;27(21):9925. doi: 10.3390/ijms27219925 (PMC13764610; doi:10.3390/ijms27219925)
Supplement: Supplementary file 1 [file ijms-27-09925-s001.zip › ijms-4594755-supplementary.pdf]

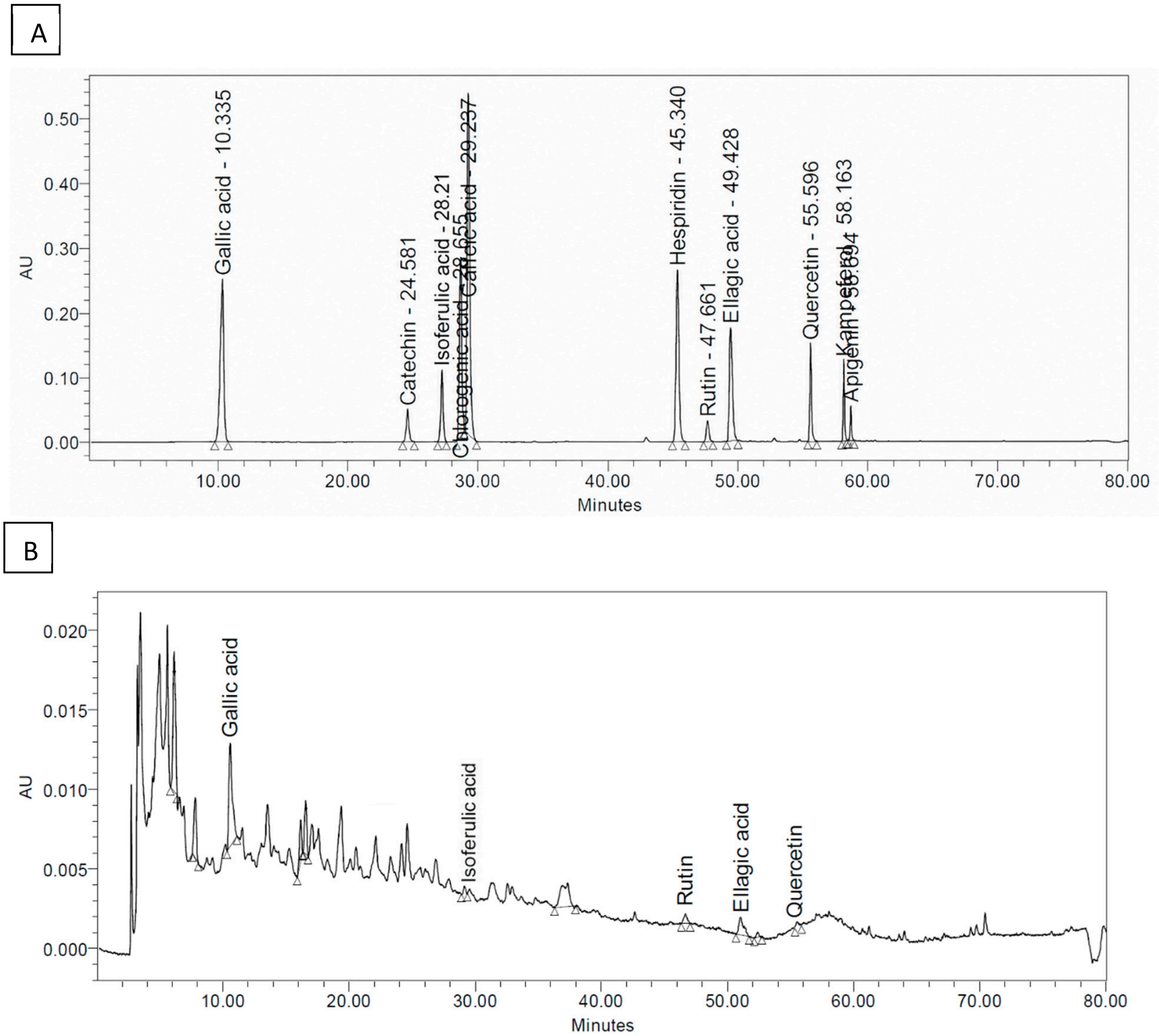

Figure S1 Targeted HPLC-PDA analysis of selected phenolic and flavonoid compounds recorded at 280 nm. (A) Reference-standard mixture; (B) *T. pinoyi* CCASU-TP-03 extract

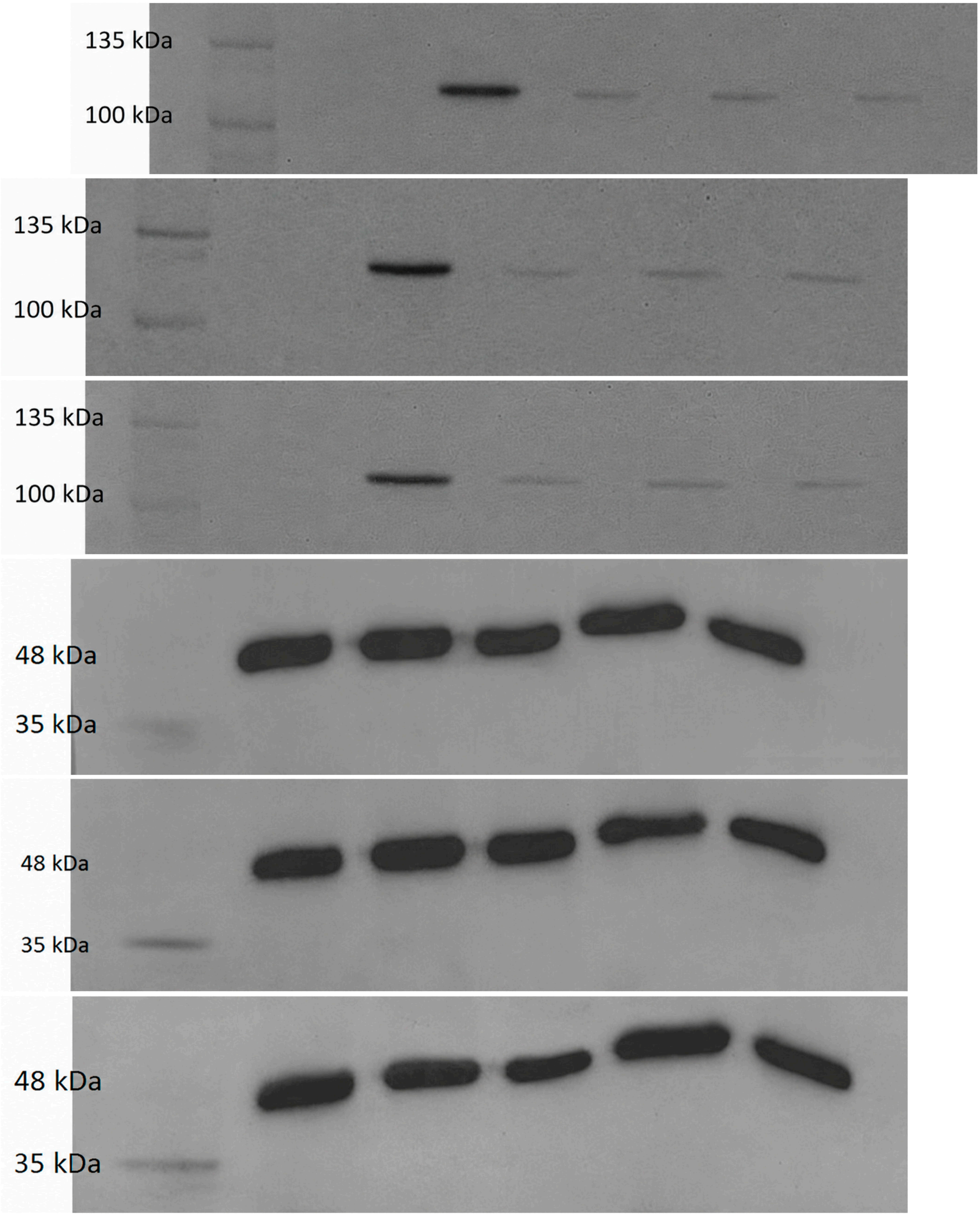
135 kDa
100 kDa
135 kDa
100 kDa
135 kDa
100 kDa
48 kDa
35 kDa
48 kDa
35 kDa
48 kDa
35 kDa

4. Results

❖ Western blot raw images

NOS2	β-Actin
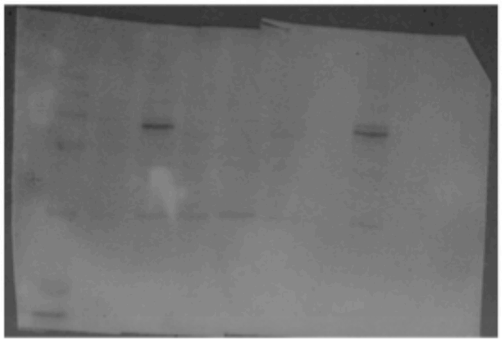	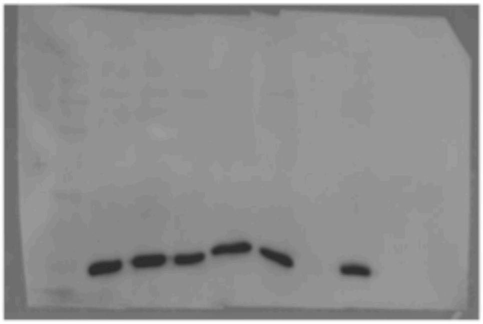

Address: HQ: 8 Jan van Goyenkade, 1075HP Amsterdam, Netherlands. Labs: Al-Mokattam mall, street 9, 2nd floor, AlMokattam, Cairo, Egypt.
Phone: +201064488216.
www.nawah-scientific.com

Figure S2. Replicates and uncropped Western blot analysis of iNOS expression in RAW264.7 macrophages following treatment with *T. pinoyi* CCASU-TP-03 extract.

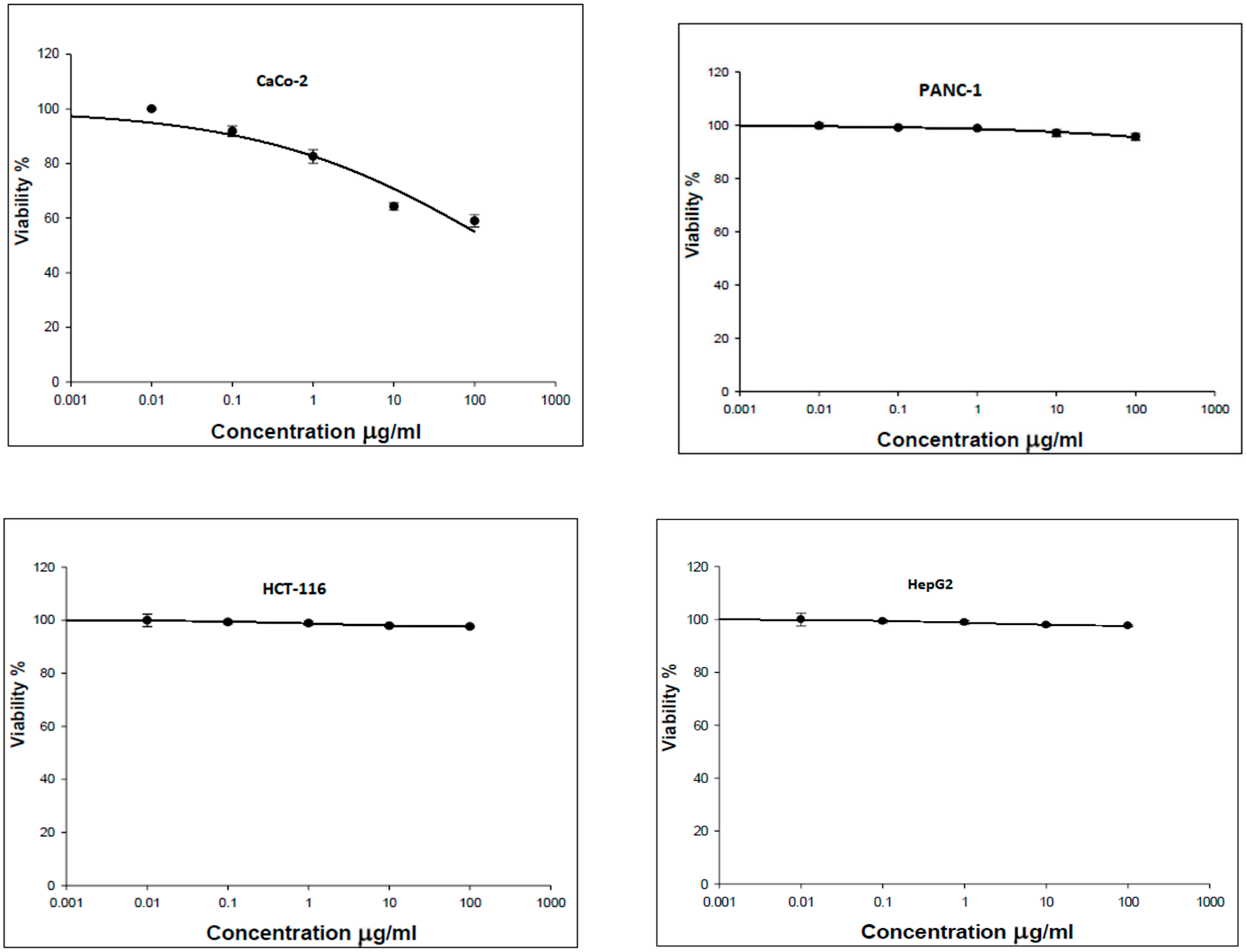

Figure S3. Concentration–response curves showing the effect of *T. pinoyi* CCASU-TP-03 extract on the viability of CaCo-2, PANC-1, HCT-116, and HepG2 cells. Cells were exposed to increasing concentrations of the extract ranging from 0.01 to 100 µg/mL, and cell viability was determined using the sulforhodamine B (SRB) assay and expressed relative to untreated control cells.
